# M2 Macrophage Polarization Mediated by Complement C3 from Hedgehog-Activated Fibroblasts Establishes an Immunosuppressive Niche in Gastric Cancer

**DOI:** 10.3390/cancers17193164

**Published:** 2025-09-29

**Authors:** Jiaheng Lou, Jingcheng Zhang, Zhiyuan Song, Shuo Zhang, Sicheng Zhao, Yunhai Wei, Guiping Chen, Tao Jiang, Guangji Zhang

**Affiliations:** 1 School of Basic Medical Sciences, Zhejiang Chinese Medical University, Hangzhou 310053, China; ljh990704@163.com (J.L.);; 2 Zhejiang Key Laboratory of Blood-Stasis-Toxin Syndrome, Zhejiang Chinese Medical University, Hangzhou 310053, China; 3 Huzhou Central Hospital, Huzhou 313000, China; 4 Zhejiang Provincial Hospital of Chinese Medicine, Hangzhou 310006, China; 5 Traditional Chinese Medicine “Preventing Disease” Wisdom Health Project Research Center of Zhejiang, Hangzhou 310053, China

**Keywords:** gastric cancer, hedgehog signaling pathway, fibroblast, macrophage, T cell, single-cell RNA-seq, tumor immunosuppressive microenvironment

## Abstract

**Simple Summary:**

Aberrant activation of the Hedgehog signaling pathway plays a critical regulatory role in the pathological progression of various cancers. In this study, we successfully identified a fibroblast subset with a highly activated Hh signaling pathway—MMP1-positive fibroblasts—using single-cell sequencing technology. Furthermore, through integrated multi-omics analyses combined with in vitro and in vivo experiments, we revealed that this subset possesses the function of specifically secreting complement C3 protein: it can induce the polarization of M2-type macrophages, remodel the tumor immunosuppressive microenvironment, and ultimately, accelerate the proliferation and metastasis of gastric cancer cells. This study uncovers a novel molecular mechanism by which aberrant activation of the Hh pathway drives gastric cancer progression through the MMP1-positive fibroblast–C3–macrophage axis, providing a potential therapeutic strategy for targeting the tumor immunosuppressive microenvironment.

**Abstract:**

**Introduction**: The Hedgehog (Hh) signaling pathway is aberrantly activated in various types of cancer and plays a critical regulatory role. However, its biological significance in gastric cancer remains unclear. In this study, the mechanism underlying the role of Hh in gastric cancer progression and prognosis was investigated through bioinformatics analysis as well as in vitro and in vivo experiments. **Methods**: In this study, a systematic analysis of scRNA-seq datasets and bulk RNA-seq datasets from gastric cancer patients derived from the GEO database and TCGA database was performed by us, which revealed the activation characteristics of Hh in different cell types within the gastric cancer tumor microenvironment (TME). Furthermore, through conducting multiplex immunofluorescence staining experiments on clinical gastric cancer samples, we clarified the association mechanism between fibroblasts with highly activated Hh and the gastric cancer tumor immunosuppressive microenvironment. Finally, by means of in vitro and in vivo experiments, we elucidated the key molecular mechanism by which fibroblasts with highly activated Hh remodel the gastric cancer tumor immunosuppressive microenvironment. **Results**: We identified a distinct subpopulation of fibroblasts, designated MMP1 + FIB, in the gastric cancer tumor microenvironment. Studies revealed that this subpopulation can significantly activate Hh, suggesting it may play a crucial role in the regulation of the TME. Subsequent mechanistic investigations further confirmed that MMP1 + FIB exhibits a significant correlation with the immunosuppressive state of the TME (R = 0.29, *p* = 2.5 × 10^−0.8^). In terms of the specific functions, the complement system in this fibroblast subpopulation is significantly activated (*p* < 0.05); further studies demonstrated that MMP1 + FIB can induce the polarization of macrophages toward the M2 subtype (an immunosuppressive phenotype) by specifically secreting complement C3 protein. Collectively, these processes contribute to the establishment of an immunosuppressive TME and ultimately promote the proliferation and metastasis of gastric cancer cells. **Discussion**: Aberrant activation of the Hh signaling pathway promotes gastric cancer progression via the MMP1 + FIB–C3–macrophage axis, providing a potential therapeutic strategy for targeting the tumor microenvironment.

## 1. Introduction

Gastric cancer (GC) ranks as the fifth most prevalent malignancy globally and the fourth principal cause of cancer-related deaths, and it has a dismal five-year survival rate of only 10%, with clinical management facing significant challenges [1]. This predicament stems from a complex etiological network driven by gastric cancer’s heterogeneity, where drivers such as oncogenes in malignant GC subtypes, *Helicobacter pylori*-induced chronic inflammation, and reprogramming of the tumor microenvironment (TME) collectively shape disease progression [2,3,4]. Emerging single-cell and spatial transcriptomics technologies are systematically dissecting the cellular ecology of the TME, illuminating how cancer-associated fibroblasts (CAFs), myeloid-derived suppressor cells (MDSCs), and spatially organized cytokine networks synergistically establish an immunosuppressive ecosystem [5]. Notably, CAFs have been validated as central regulators of immune exclusion through matrix remodeling and metabolic reprogramming, yet their subtype-specific functions in gastric cancer remain undefined [6].

The Hedgehog (Hh) signaling pathway, a core regulator of embryonic development and tissue homeostasis, has been shown to be aberrantly activated—defined as the abnormal and sustained activation of its key molecules and downstream signaling—in tumorigenesis and progression across multiple cancer types [7,8]. In basal cell carcinoma and medulloblastoma, the oncogenic mechanisms of Hh are well-defined, with >90% of cases harboring PTCH1 or SMO gene mutations that directly drive constitutive activation of the canonical ligand–receptor–GLI signaling cascade [9]. However, in solid tumors such as prostate cancer, breast cancer, and gastric cancer, Hh exhibits highly context-dependent roles: it forms dynamic crosstalk with WNT/Notch pathways via non-canonical mechanisms (e.g., KRAS/MAPK-mediated stabilization of GLI1 protein), while mesenchymal–epithelial bidirectional communication through paracrine Hh ligand secretion in the TME sustains the self-renewal of cancer stem cells (CSCs) [10]. Notably, although preclinical studies suggest that Hh targeting inhibits GC progression, clinical trials of SMO inhibitors (e.g., Vismodegib) have shown no significant survival benefit in GC patients, implying that Hh may drive tumor immune microenvironment (TIME) remodeling through cell-type-specific mechanisms in GC, rather than relying on canonical tumor cell autonomous activation. Deciphering the regulatory network of Hh in GC stromal–immune crosstalk therefore represents a critical breakthrough for overcoming bottlenecks in targeted therapy.

With the continuous advancement of biotechnology, large-scale multi-omics datasets—including genomics, transcriptomics, epigenomics, and others—have been generated to systematically and comprehensively measure tumor characteristics across different molecular levels. Collectively, these datasets constitute a foundational resource for comprehending the molecular architecture of cancer. Accordingly, the proliferation of omics data has prompted the advancement of a wide array of bioinformatics resources to facilitate the management and interpretation of large-scale biological datasets. The integration and computational analysis of multi-omics data have transformed cancer research, enabling the elucidation of molecular processes underlying tumor development, characterization of the tumor microenvironment (TME), and discovery of biomarkers for early diagnosis and prognostic assessment, and informing the design of tailored targeted treatments. For example, applications of single-cell RNA sequencing (scRNA-seq) in dissecting the tumor microenvironment have yielded critical biological insights, including tumor heterogeneity, dynamics, and roles in disease progression and responses to immune checkpoint inhibitors and other immunotherapies [11]. Spatial transcriptomics, with its unbiased capacity for spatial molecular profiling, has successfully constructed spatial molecular atlases of multiple organs. This technology not only systematically reveals the three-dimensional molecular architecture of normal tissues but also precisely captures dynamic disruptions in tissue microstructure under pathological states [12] (e.g., tumor infiltration). Particularly in oncology, spatial transcriptomics has unveiled spatial coupling patterns between malignant clonal evolution and the formation of immunosuppressive microenvironments by deciphering molecular gradient signatures at the cancer nest–stroma interface, providing a novel perspective for overcoming treatment bottlenecks in solid tumors [13]. In this study, single-cell sequencing data were integrated with spatial transcriptomics and other methodologies to investigate the potential biological functions of Hh in gastric cancer., aiming to deepen our understanding of GC tumor heterogeneity and identify new therapeutic strategies.

## 2. Materials and Methods

### 2.1. Collection of Single-Cell Datasets and RNA-Seq Datasets

To acquire both transcriptomic and clinical information, the research team retrieved publicly accessible data from two major sources: the Cancer Genome Atlas (TCGA) and the Gene Expression Omnibus (GEO) [14]. The TCGA-STAD cohort comprised transcriptomic and clinical data from 438 gastric cancer (GC) patients. Genomic expression profiles quantified in FPKM, along with the corresponding clinical information, were retrieved as of 8 May 2022. For consistency across datasets, the FPKM values were converted to transcripts per million (TPM). Additional expression data were obtained from the GEO, including the GSE163558 dataset (10 samples, GC) [15], the GSE183904 dataset (40 samples, GC) [16], and the GSE203612 dataset (GC, spatial transcriptomics) [17].

### 2.2. Raw Data Processing and Quality Control

The GSE163558, GSE183904, and GSE203612 datasets were downloaded from the GEO database. Data quality control and preprocessing of scRNA-seq data were conducted using the Seurat package in R software (version 5.2.1), aiming to ensure the reliability and usability of subsequent analytical results [18]. Three quality control measures were applied with the following exclusion criteria: (1) the number of detected genes per cell was between 200 and 2500, (2) the percentage of mitochondrial genes in cells was below 20%, and (3) the total number of RNA molecules detected per cell was less than 10,000.

### 2.3. Data Integration, Unsupervised Clustering, and Cell Type Annotation

Principal component analysis (PCA) was carried out on the integrated expression matrix via the RunPCA function. The first 15 principal components (PCs) derived from this analysis were subsequently used as input for the FindNeighbors function. The resolution parameter of the FindClusters function was set to 0.2 for the initial execution and 0.1 for subsequent runs. GO and KEGG functional annotation and enrichment analyses of DEGs were conducted using the clusterProfiler package (v4.14.4) [19], with the GO and KEGG enrichment annotations requiring a false discovery rate (FDR) < 0.05.

### 2.4. Single-Cell Trajectory Analysis

To explore the potential lineage differentiation among malignant cell populations, trajectory analysis was performed using the monocle3 algorithm (v1.4.13) [20]. The CellDataSet object was generated via the newCellDataSet function. Cell trajectories were determined using the orderCells function and visualized using the plot_cells function.

### 2.5. Intercellular Crosstalk Network Analysis

CellChat was employed to investigate the molecular interaction networks between distinct cell types. Using CellChat, ligand–receptor pairs exhibiting a *p*-value below 0.05 were identified as statistically significant interactions among distinct cell subtypes.

### 2.6. Genomic Variation Analysis

Gene Set Variation Analysis (GSVA) was implemented using the GSVA package (v2.0.5) [21]. This non-parametric, unsupervised method transformed the classical gene matrix (genes × samples) into a gene set enrichment matrix, generating enrichment scores for each sample and pathway. The GSVA matrix was then clustered using the “Pheatmap” package (v1.0.12) and visualized as a heatmap.

### 2.7. Gene Set Scoring

Gene set scoring was performed using the R package irGSEA (v3.3.2) [22], which evaluates the performance of multi-gene set scoring methods applied to scRNA-seq data, including AUCell, UCell, singscore, ssGSEA, JASMINE, and Viper. Additionally, the AUCell algorithm was used to calculate the expression of MMP1 + FIB-related genes, Hh-related genes, and other gene sets in single-cell transcriptome samples.

### 2.8. GSEA for KEGG Enrichment Analysis

GSEA was performed to identify biological pathways enriched in gastric cancer associated with Hh-related core gene scores (Hh.score). Patients were stratified into the high and low Hh.score groups using the median Hh.score as the cutoff. GSEA was then conducted to compare these two groups.

### 2.9. Differential Expression Analysis

Differential expression analysis was performed using the limma package (v3.62.2) [23] in R to identify differentially expressed genes (DEGs) in the high and low Hh.score groups, respectively. DEGs in TCGA-STAD were independently filtered with thresholds of |log_2_FC| > 1 and *p*-value < 0.05.

### 2.10. Machine Learning Analysis

Signature-related genes of MMP1 + FIB were screened, and multiple predictive models for clinical prognosis prediction in gastric cancer patients were constructed and evaluated using the Mime1 package (v0.0.0.9) [24] via 101 machine learning algorithm combinations for comprehensive analysis.

### 2.11. Nomogram Construction and Independent Prognostic Analysis

To assess the independence of the risk score while accounting for clinical variables and survival outcomes, both univariate and multivariate Cox regression analyses were employed. Kaplan–Meier survival curves, including cumulative event tables and log-rank tests, were plotted using the survminer (v0.5.0) and survival (v3.5.5) R packages.

### 2.12. Multiplex Immunofluorescence

Multiplex Immunofluorescence (mIHC) was performed using the PANO Multiplex IHC Kit (10234100100; Panovue, Beijing, China) according to the manufacturer’s instructions. Commercially purchased GC tissue microarrays (TMAs; YEPCOME Biotechnology Co., Shanghai, China) were used in this study, with the TMA sections comprising three GC tissues. A total of 60 cores with a diameter of 1.5 mm were arrayed in paraffin blocks within the TMA. Briefly, GC tissue microarrays were incubated overnight in a hot air oven at 65 °C. They were then dewaxed three times in fresh xylene for 10 min each, rehydrated through a gradient series of ethanol (100% to 95% to 70%), and washed three times with PBS. Antigen retrieval was performed via microwave heating, followed by cooling in an ice water bath for at least 15 min. The TMA sections were incubated with blocking solution (0018001120; Panovue, Beijing, China) at room temperature for 15 min, followed by primary antibody incubation for 30 min, secondary antibody incubation for 10 min, and staining with TSA Opal fluorophores for 10 min. These steps (antigen retrieval, blocking, primary antibody incubation, and TSA Opal staining) were repeated for each marker. Finally, the TMA sections were counterstained with DAPI (D9542; Sigma-Aldrich, St. Louis, MO, USA) for 5 min and mounted with coverslips. Whole TMA section imaging was performed using a panoVIEW vs. 200 imaging system (Panovue, Beijing, China). The primary antibodies used were CD3 (1:500; AF20162, AF), CD8 (1:500; AF20211, AF), α-SMA (1:2000; AFMM002, AF), and GLI1 (1:500; 2534S, PTG). Multiple immunofluorescence images were analyzed using ImageJ (Fiji, version 1.54).

### 2.13. Animal Model Construction

Nude mice (4–6 weeks old, *n* = 10) were purchased from Shanghai Slac Laboratory Animal Co., Ltd. (Shanghai, China) For the subcutaneous xenograft experiments, mice were randomly assigned to the experimental groups (*n* = 5) for study purposes. After a one-week adaptive feeding period, mice in the C3 group were subcutaneously injected with C3 recombinant protein at a dose of 20 μg/kg, administered every other day for one week, while control group mice received subcutaneous injections of an equal volume of physiological saline. GC MKN-45 cell lines were subcutaneously implanted into the lateral flanks of the mice. Tumors were evaluated every two days, and the tumor volume was estimated using the formula (length × width^2^/2). After two weeks, the mice were euthanized, and the tumor weights were assessed.

### 2.14. Flow Cytometry

Tumor tissue samples were mechanically disaggregated and digested to generate single-cell suspensions. Subsequently, red blood cells were effectively eliminated from the resulting suspensions by treatment with a specialized lysing buffer. Cells were pre-stained with specific antibodies against mouse CD45 (S0B1656-100T; STARTER, Hangzhou, China), CD11b (abs1850015-100T; Absin, Shanghai, China), F4/80 (565410; BD Pharmingen, Franklin Lakes, NJ, USA), CD86 (560582; Absin, Shanghai, China), and CD206 (568808; BD Pharmingen, Franklin Lakes, NJ, USA). Further, live/dead cell staining was performed using DAPI (564907; BD Pharmingen, Franklin Lakes, NJ, USA). Labeled cells were washed, and samples were analyzed using a CytoFLEX S flow cytometer (Beckman, Brea, CA, USA).

### 2.15. Colony Formation Assay

Cells were plated in 6-well plates at a density of 1 × 10^3^ cells per well and cultured for 14 days, with the medium refreshed every three days. Following the culture period, cell colonies were fixed in 4% paraformaldehyde for 15 min, stained with Giemsa solution for 20 min, and then imaged for documentation and quantitative analysis.

### 2.16. Cell Viability Assay

For the CCK-8 assay, cells were seeded into 96-well plates at a density of 5 × 10^3^ cells per well. The model group was treated with conditioned medium from RAW264.7 cells stimulated with C3 recombinant protein. After 24 h, 100 μL of medium containing 10% CCK-8 reagent was added to each well, and the plates were incubated for an additional 2 h. The optical density (OD) of each well was measured at 450 nm at different time points to assess the cell viability.

### 2.17. Statistical Analysis

All the data analyses were performed using the R platform (specifically version 4.4.3). For comparing continuous variables between two subgroups, *t*-tests or Wilcoxon rank-sum tests were applied. Differences among three groups were evaluated using one-way ANOVA or Kruskal–Wallis tests. Pearson correlation was used to assess correlations between normally distributed variables, while Spearman correlation was employed for non-normally distributed variables. To control for the inflation of type I errors in multiple testing scenarios, the Benjamini and Hochberg (BH) method was utilized to estimate the false discovery rates (FDR), with adjusted *p*-values accounting for the number of tests performed. For survival analysis, Kaplan–Meier analysis and log-rank tests were conducted using the “survminer” R package to evaluate the survival differences between groups. Implementation of the BH method for FDR control ensured robust and reliable results, minimizing the likelihood of false positives from multiple comparisons.

## 3. Results

### 3.1. Hedgehog Signaling Pathway Is Significantly Activated in Fibroblasts of the Tumor Microenvironment of Gastric Cancer

**Figure 1:**
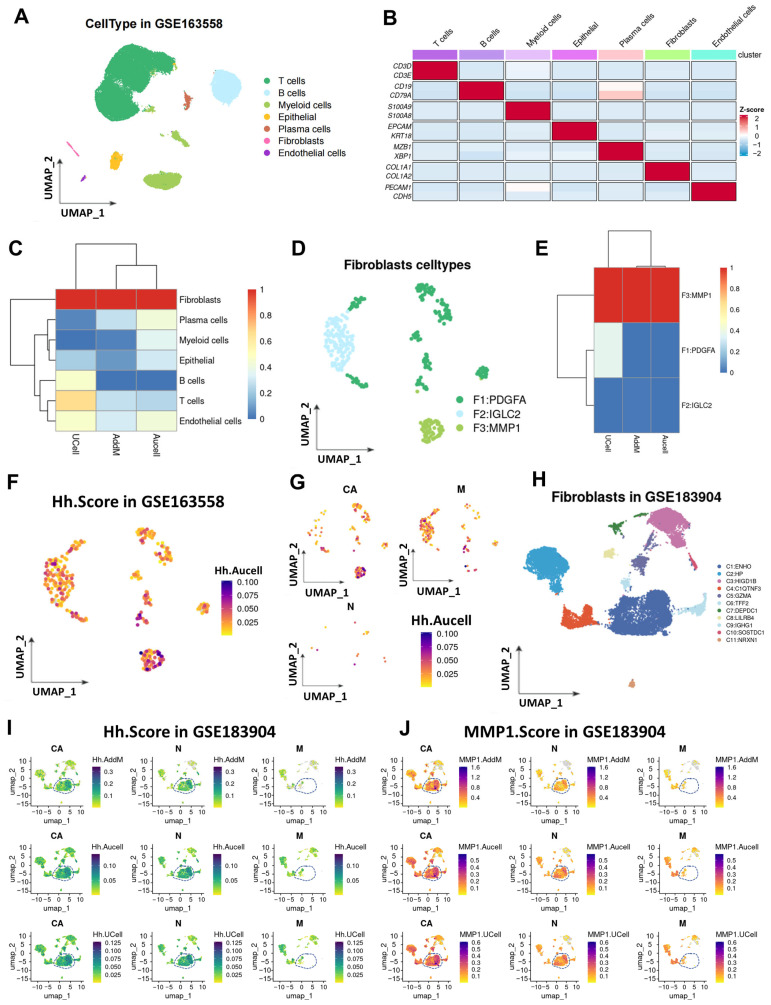
Activation levels of the Hh pathway in different cell subsets within the gastric cancer tumor microenvironment. (**A**) UMAP shows the distribution of seven cell types in the GSE163558 dataset. (**B**) Marker genes of each cell type. (**C**) Heatmap presenting the activation levels of the Hh gene set in the seven cell subsets based on three enrichment analysis methods (UCell, AddModuleScore, and Aucell). (**D**) UMAP shows the distribution of three subsets of fibroblasts after secondary clustering. (**E**) Heatmap presenting the activation levels of the Hh gene set in the three fibroblast subsets based on three enrichment analysis methods (UCell, AddModuleScore, and Aucell). (**F**) UMAP shows the activation level of the Hh gene set in fibroblasts. (**G**) UMAP shows the activation status of the Hh pathway in fibroblast populations from samples at different lesion stages (carcinoma in situ, paracancerous tissues, and metastatic tumors). (**H**) UMAP shows the subset distribution of fibroblasts from samples at different lesion stages (carcinoma in situ, paracancerous tissues, and metastatic tumors) in the GSE183904 dataset after secondary dimensionality reduction and clustering. (**I**) UMAP shows the activation status of the Hh gene set in fibroblast populations from samples at different lesion stages in the GSE183904 dataset. (**J**) UMAP shows the activation status of the top 50 differentially expressed genes in the MMP1 + FIB subset among fibroblast populations from samples at different lesion stages in the GSE183904 dataset. CA, gastric cancer; N, normal gastric tissue; M, metastatic tumor.

In this study, the GSE163558 dataset was retrieved from the GEO database, which contains 10 single-cell samples (primary tumors, adjacent tissues, and metastatic tumors from different organs/tissues) with a total of 53,940 cells. Unsupervised clustering preprocessing was performed using the Seurat package (Supplementary Figure S1A,B), and 10 cell clusters were finally identified (Supplementary Figure S1C). Based on specific gene signatures, manual annotation of these 10 cell subsets was conducted (Supplementary Figure S1D). According to the results of the manual annotation, the aforementioned cells were divided into seven subsets, namely T cells, B cells, myeloid cells, epithelial cells, plasma cells, fibroblasts, and endothelial cells (Figure 1A), and the marker genes of each subset were also labeled (Figure 1B). To explore the expression characteristics of Hh-related genes in the gastric cancer microenvironment, three gene enrichment methods (UCell, AddModuleScore, and Aucell) were used in this study to calculate the activation level of the Hh gene set in all cells. All three enrichment analysis methods showed that among the seven cell types in the gastric cancer microenvironment, fibroblasts had the highest activation level of the Hh pathway (Figure 1C, Supplementary Figure S2F). Given that fibroblasts are a core component of the tumor microenvironment (TME) and play a key regulatory role in tumor progression, this study further analyzed the heterogeneity in the activation level of the Hh pathway among different fibroblast subsets. Fibroblasts were extracted for secondary dimensionality reduction, and this cell population was divided into three subsets based on their major differentially expressed genes (Supplementary Figure S1E), which were named PDGFA-positive fibroblasts (PDGFA + FIB), IGLC2-positive fibroblasts (IGLC2 + FIB), and MMP1-positive fibroblasts (MMP1 + FIB) (Figure 1D). The three gene enrichment analysis methods (UCell, AddModuleScore, and Aucell) were again used to detect the activation level of the Hh gene set in these three fibroblast subsets, and it was found that MMP1 + FIB had the highest Hh activation level (Figure 1E,F). Furthermore, it was observed that compared with adjacent tissues and peritoneal metastatic tissues of gastric cancer patients, MMP1 + FIB was significantly enriched in the TME of primary lesions (Figure 1G). These results indicate that there is a subset of fibroblasts with significantly activated Hh in the gastric cancer TME. To verify this conclusion, an external validation was conducted in the GSE183904 dataset. Using unsupervised clustering analysis in the Seurat package, a total of 11 cell clusters were identified and manually annotated into seven different cell subsets (Supplementary Figure S2A–D). Consistently, the highest activation level of Hh was also found in fibroblasts (Supplementary Figure S2E). Moreover, among the fibroblast subsets obtained by secondary clustering, there was a fibroblast subset with significantly activated Hh, and this subset was highly similar to the aforementioned MMP1 + FIB (Figure 1I,J).

### 3.2. The Relationship Between MMP1 + FIB and the Malignant Progression of Gastric Cancer

**Figure 2:**
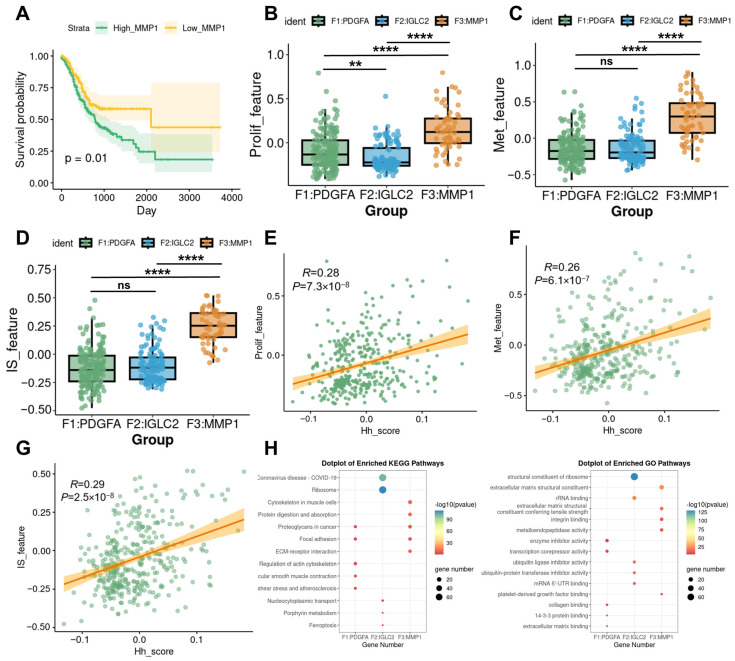
Association of MMP1 + FIB with malignant progression of gastric cancer. (**A**) Survival analysis curves of patients in the High_MMP1 and Low_MMP1 groups. (**B**–**D**) Inter-group comparison analysis showing differences among fibroblast subsets in terms of tumor proliferation, metastasis, and immunosuppression. (**E**–**G**) Correlation analysis revealing associations between the Hh score and tumor proliferation, metastasis, and immunosuppression. (**H**) Results of KEGG and GO functional annotation analyses for each fibroblast subset. Hh_score, gsva enrichment score of the Hedgehog signaling pathway gene set; Prolif_feature, gsva enrichment score of the tumor proliferation gene set; Met_feature, gsva enrichment score of the tumor metastasis gene set; IS_feature, gsva enrichment score of the immunosuppressive gene set. ns, *p* ≥ 0.05; ** *p* ≤ 0.01; **** *p* ≤ 0.0001.

To further explore the association of MMP1 + FIB with the malignant progression of gastric cancer, we screened the top 100 marker genes of MMP1 + FIB using the FindAllMarkers function. Based on the results of the Gene Set Variation Analysis (GSVA) enrichment analysis calculated from the TCGA-STAD dataset, patients were divided into two groups: the high MMP1 + FIB signature score group (High_MMP1) and the low MMP1 + FIB signature score group (Low_MMP1), according to the optimal cutoff value of the signature score. We found that patients in the High_MMP1 group had shorter overall survival (Figure 2A). Based on the existing literature, this study further compiled gene sets associated with key biological processes mediated by fibroblasts, including tumor proliferation, metastasis, immunosuppression, immune activation, and inflammatory chemotaxis. The AddModuleScore method was used to calculate the enrichment scores of each of these 10 gene sets group by group. Inter-group comparison analysis showed that MMP1 + FIB significantly promoted the proliferation, metastasis, and immunosuppression of gastric cancer (Figure 2B–D, Supplementary Figure S3A–G). The results of the correlation analysis were consistent with the above findings (Figure 2E–G; Supplementary Figure S3H–N). After functional annotation of each fibroblast subset, it was found that MMP1 + FIB was significantly enriched in functional pathways such as extracellular matrix structural components, integrin binding, collagen binding, metalloendopeptidase activity, and ECM–receptor interaction. These functions were not observed in other fibroblast subsets (Figure 2H). These findings suggest that MMP1 + FIB may be involved in regulating the structure and function of the extracellular matrix as well as related pathological processes. Recent studies have shown that extracellular matrix (ECM) components secreted by fibroblasts (such as collagen and laminin) can promote tumor stromal sclerosis, enhance the mechanical barrier, and help tumors evade immune recognition [25,26,27]. These results confirm that MMP1 + FIB, characterized by abnormal activation of the Hh signaling pathway, inhibits anti-tumor immune responses and is closely associated with the proliferation and metastasis of gastric cancer.

### 3.3. Differentiation Trajectory and Related Bioinformatics Characteristics of MMP1 + FIB

**Figure 3:**
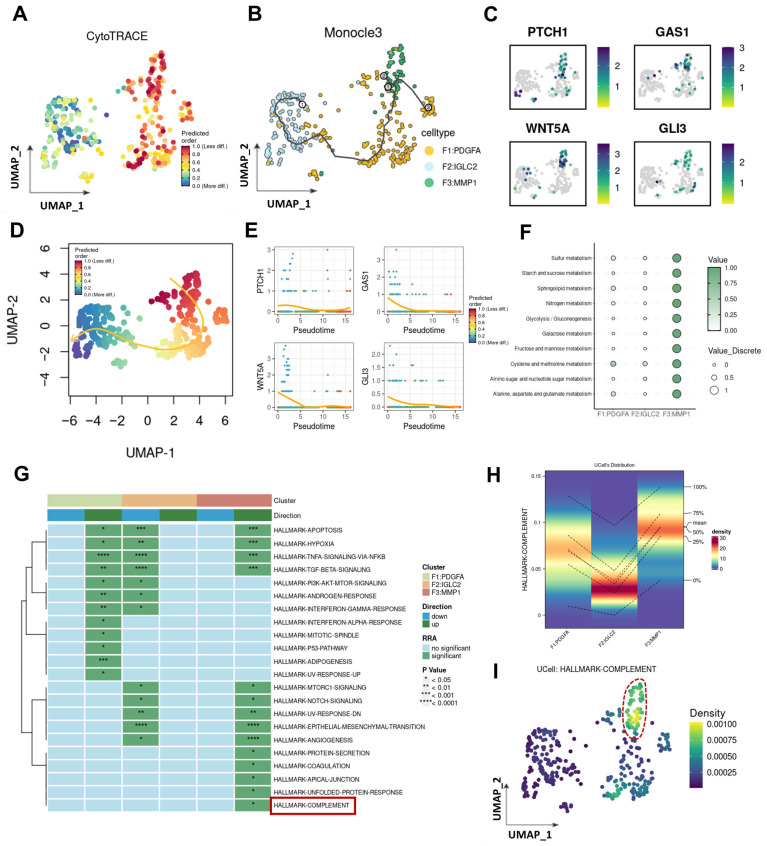
Lineage differentiation dynamics and molecular characteristics of the MMP1 + FIB regulatory network. (**A**) CytoTRACE analysis reveals the differentiation levels of each fibroblast subset. (**B**) Monocle trajectory analysis deduces the lineage differentiation structure and sequence of fibroblast subsets. (**C**) UMAP shows the expression profiles of Hh core genes (PATCH1, GAS1, WNT5A, GLI1) in different fibroblast subsets. (**D**) Slingshot trajectory analysis deduces the lineage differentiation structure and sequence of fibroblast subsets. (**E**) Expression profiles of Hh core genes (PATCH1, GAS1, WNT5A, GLI1) in different differentiation stages. (**F**) ScMetabolism analysis reveals the metabolic patterns of the three fibroblast subsets. (**G**) irGSEA analysis reveals the pathway activation statuses of the three fibroblast subsets. (The area marked by the red box is exactly the Complement pathway as described in the text.) (**H**) Activation status of the complement system in each fibroblast subset. (**I**) UMAP shows the activation status of the complement system in fibroblasts. (The fibroblasts marked by the red box are exactly those with the highest activation level of the Complement pathway).

Given that MMP1 + FIB is the fibroblast subset with the highest activation level of the Hh pathway, this study further investigated its differentiation trajectory, bioinformatics characteristics, and Hh activation status. CytoTRACE analysis revealed that MMP1 + FIB exhibits a low degree of differentiation, which indicates that this cell subset possesses strong stemness characteristics and a relatively complex gene expression pattern, and it is typically found in the initial stage of the cell differentiation process. Therefore, in the Monocle3 pseudotime analysis, we designated MMP1 + FIB as the starting point of differentiation, and we found that the expression levels of Hh-related genes gradually decreased as cells differentiated (Figure 3B,C, Supplementary Figure S4A–E). To further validate the above conclusion, this study employed the slingshot tool to infer the lineage differentiation structure and sequence of fibroblasts. The results obtained were consistent with the aforementioned findings: MMP1 + FIB localizes at the initial stage of the cell differentiation trajectory, and as cells differentiate, the expression levels of Hh-related genes also showed a gradual decreasing trend (Figure 3D,E). We further explored the bioinformatics characteristics of MMP1 + FIB. Results of the ScMetabolism analysis based on the KEGG database demonstrated that the main metabolic patterns of MMP1 + FIB include glucose metabolism, starch and sucrose metabolism, butyrate metabolism, nitrogen metabolism, glycolysis/gluconeogenesis, galactose metabolism, fructose and mannose metabolism, and other metabolic pathways (Figure 3F). Furthermore, results of the irGSEA analysis showed that a total of 14 signature gene sets in MMP1 + FIB exhibited an upregulated trend, among which 5 gene sets (e.g., the Complement system) were specifically activated in this subset (Figure 3G–I).

### 3.4. CellChat Analysis Reveals Intercellular Communication Patterns of Each Fibroblast Subset in the Gastric Cancer Microenvironment

**Figure 4:**
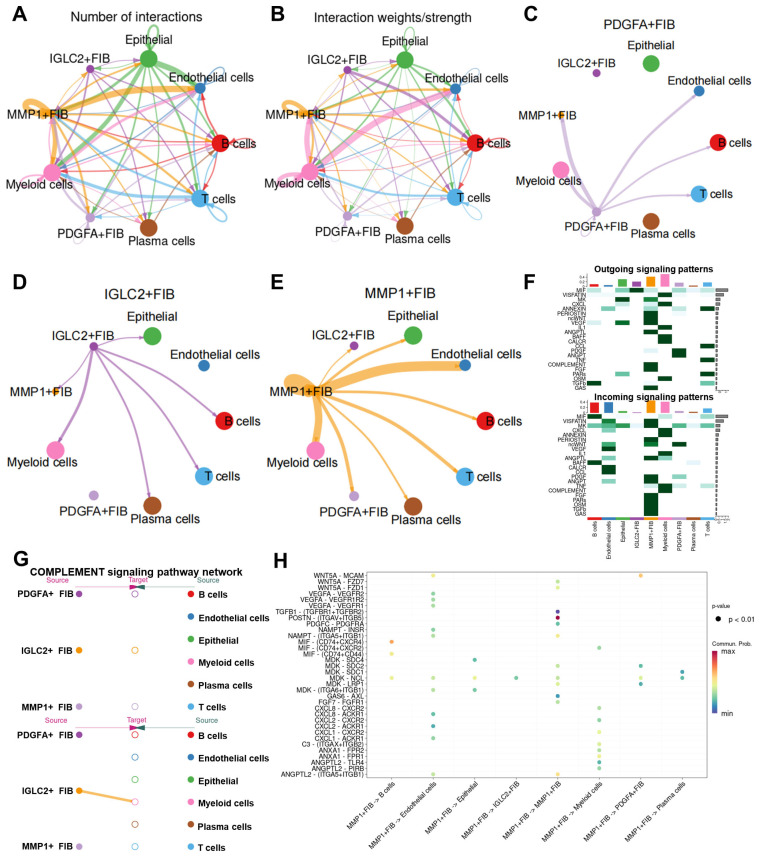
CellChat reveals cell–cell communication roles of MMP1 + FIB in the gastric cancer TME. (**A**,**B**) Circos plots depict the number and strength of the interactions among different cell types. Thicker lines indicate greater interaction numbers and stronger intensity between cell types. (**C**–**E**) Cell–cell communication networks for MMP1 + FIB, PDGFA + FIB, and IGLC2 + FIB. (**F**) Heatmap showing the relative strength of each signaling pathway network per cluster, including incoming and outgoing signaling. Color gradients reflect scaled interaction strength. (**G**) Hierarchy plot illustrating the signaling cascade of the COMPLEMENT signaling pathway network. (**H**) Bubble plot highlighting the dominant signaling pathways in MMP1 + FIB. Each dot represents a ligand–receptor pair, with the size determined by the pathway-enrichment *p*-value and color indicating the communication probability. FIB, fibroblast.

We further investigated MMP1 + FIB’s functions using CellChat to evaluate the cell–cell communication networks among the high MMP1 + FIB, PDGFA + FIB, IGLC2 + FIB, and other cell types. Cell communication analysis revealed that compared with PDGFA + FIB and IGLC2 + FIB, the count and intensity of the interactions between MMP1 + FIB and other cells were higher (Figure 4A,B). MMP1 + FIB primarily engaged with endothelial and myeloid cells (Figure 4C–E, Supplementary Figure S5A–F). This result verified our conjecture that MMP1 + FIB can reconstruct the tumor immune microenvironment. Therefore, we delved deeply into the effect of MMP1 + FIB on myeloid cells. Heatmaps demonstrated MMP1 + FIB-specific modulation of myeloid cells via the COMPLEMENT signaling pathway (Figure 4F,G). Further analysis identified complement C3 as the dominant mediator of MMP1 + FIB–myeloid cell communication (Figure 4H). PDGFA + FIB primarily influenced B cells via the MIF signaling pathway (Supplementary Figure S5G,H), whereas IGLC2 + FIB mainly regulated MMP1 + FIB through PDGF and ANGPT signaling pathways (Supplementary Figure S5I–L). These findings demonstrate that MMP1 + FIB—fibroblasts with aberrantly activated Hh—are distinct from PDGFA + FIB and IGLC2 + FIB. Specifically, MMP1 + FIB influences myeloid cells through COMPLEMENT pathway activation and selective secretion of complement C3, suggesting a potential role in remodeling the tumor immunosuppressive microenvironment.

### 3.5. Bulk RNA-Seq Validates the Remodeling Effect of MMP1 + FIB on the Gastric Cancer TIME

**Figure 5:**
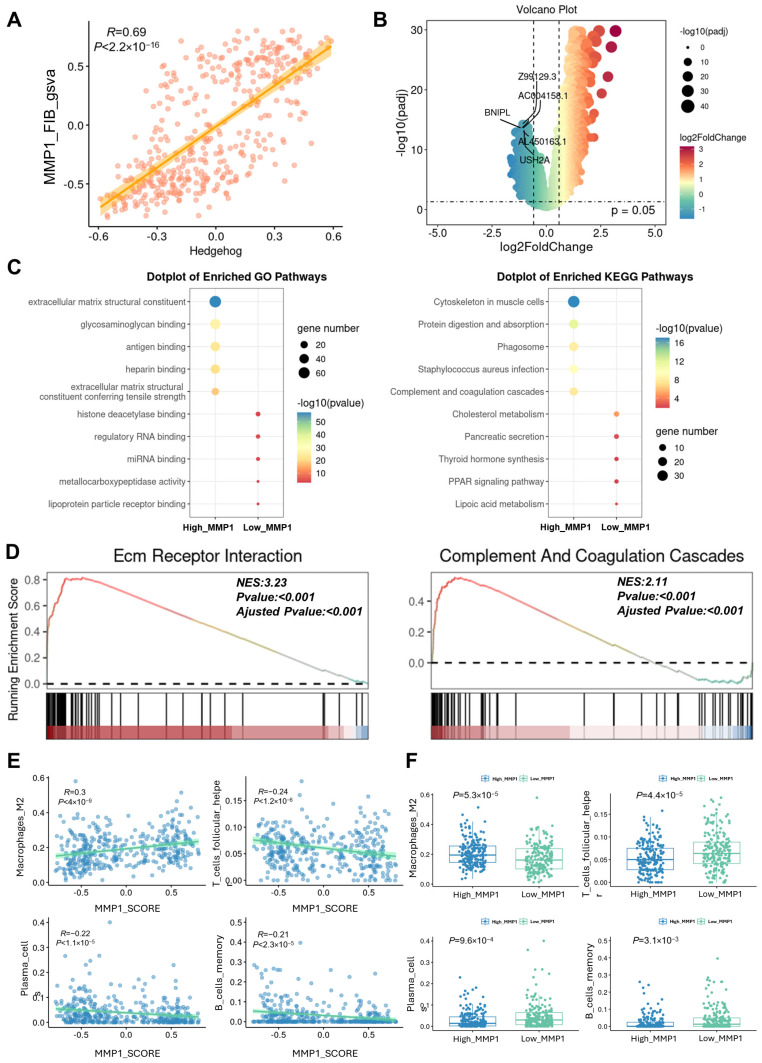
Validation of single-cell sequencing results by bulk RNA-Seq. (**A**) Correlation scatter plot showing the association between the Hh activation level and the MMP1 + FIB infiltration level in patients. (**B**) Volcano plot of the DEGs between the High_MMP1 group and the Low_MMP1 group. (**C**) Results of the GO and KEGG functional annotation analyses for the High_MMP1 group and the Low_MMP1 group. (**D**) GSEA enrichment analysis showing the activation status of the ECM–receptor interaction and complement and coagulation cascades. (**E**) Correlation between the MMP1 + FIB infiltration level and the infiltration levels of M2 macrophages, T cells, plasma cells, and other immune cells in patients. (**F**) Box plot presenting the differences in the infiltration levels of M2 macrophages, T cells, plasma cells, and other immune cells between the High_MMP1 group and the Low_MMP1 group. Hedgehog, gsva enrichment score of the Hedgehog signaling pathway gene set; High_MMP1, patients with high GSVA scores for MMP1 + FIB-related characteristic genes; Low_MMP1, patients with low GSVA scores for MMP1 + FIB-related characteristic genes.

At the bulk RNA-seq level, this study further validated the aforementioned findings at the single-cell level. The results showed that there was a statistically significant positive correlation between the infiltration level of MMP1 + FIB and the activation level of Hh in gastric cancer patients (Figure 5A). Subsequently, based on the infiltration level of MMP1 + FIB in patients, the patients were divided into the high MMP1 + FIB signature gene score group (High_MMP1) and the low MMP1 + FIB signature gene score group (Low_MMP1). Differential analysis revealed 996 differentially expressed genes (DEGs) between the two groups of patients, including 666 upregulated genes and 330 downregulated genes (Figure 5B). Through functional annotation analysis of the upregulated DEGs, we found that functions such as extracellular matrix structural components, extracellular matrix structures conferring tensile strength, and complement and coagulation cascades were significantly activated in the High_MMP1 group (Figure 5C). This study further performed Gene Set Enrichment Analysis (GSEA) on all the DEGs, and the results were consistent with those of the single-cell analysis: the extracellular matrix (ECM) and complement system were significantly activated (Figure 5D), and tumor immunosuppression-related pathways also showed a significant activation trend (Supplementary Figure S6A–I). In addition, immune infiltration analysis indicated that the infiltration level of MMP1 + FIB was significantly positively correlated with that of M2-type macrophages, while it was significantly negatively correlated with specific immune-activated cell subsets (e.g., T cells, B cells, and others) (Figure 5E,F, Supplementary Figure S7A–H). The aforementioned results at the bulk RNA-seq level were consistent with those from the single-cell RNA sequencing data, further confirming that the activation level of the Hh signaling pathway was significantly positively correlated with the infiltration level of MMP1 + FIB, and that MMP1 + FIB promotes tumor metastasis, proliferation, and tumor immune microenvironment (TIME) structural remodeling.

### 3.6. Construction of a Clinical Predictive Model for Gastric Cancer Patients with the MMP1 + FIB Signature

**Figure 6:**
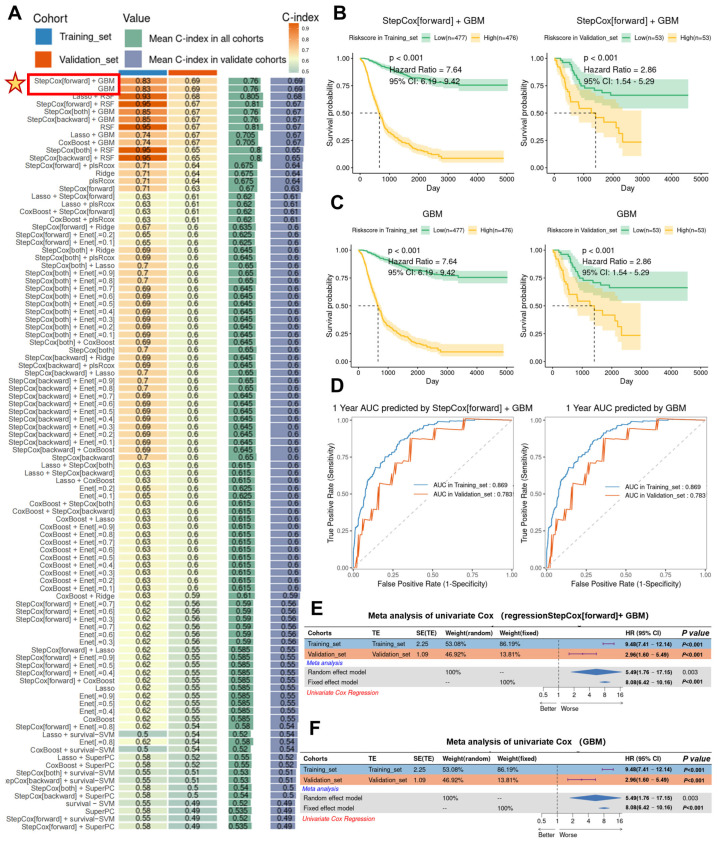
Construction of the MMP1 + FIB model and validation of its efficacy in predicting survival outcomes. (**A**) Using 10 comprehensive computational frameworks (StepCox, GBM, Lasso, RSF, plsRcox, Ridge, Enet, CoxBoost, survival-SVM, SuperPC), 101 machine learning algorithm combinations were generated. The C-index of each model was calculated in the training and validation cohorts to evaluate predictive performance. (**B**) Kaplan–Meier curves showed the association between the risk scores calculated by the StepCox[forward] + GBM combined model and patient outcomes in both the training and validation cohorts. (**C**) Kaplan–Meier curves demonstrated the relationship between the risk scores derived from the GBM model and patient outcomes in the training and validation cohorts. (**D**) ROC curves evaluated the 1-year survival area under the curve (AUC) performance of the StepCox[forward] + GBM and GBM models. (**E**) Meta-analysis of the univariate Cox regression for the MMP1 + FIB-related signature genes and survival time under the StepCox[forward] + GBM model. (**F**) Meta-analysis of the univariate Cox regression for the MMP1 + FIB-related signature genes and survival time in the GBM model.

Based on the above findings, we determined that Hh pathway activation is a hallmark of MMP1 + FIB, which are highly enriched in tumor tissues and promote tumor proliferation, metastasis, and immunosuppression—suggesting an association with poor prognosis in gastric cancer. To establish a clinical prognostic model, we first identified differentially expressed genes (DEGs) of MMP1 + FIB using FindAllMarkers and applied machine learning algorithms to analyze gastric cancer patient data. In the training cohort, we fitted 101 predictive models and evaluated their performance using the C-index on a validation dataset. The optimal models were StepCox[forward] + GBM and GBM, which achieved the highest C-index in both the training dataset (C-index = 0.83) and the validation dataset (C-index = 0.69). These models were constructed using 117 MMP1 + CAFs-related genes, with an average C-index of 0.76 across datasets (Figure 6A). Using StepCox[forward] + GBM, we calculated the MMP1 + CAFs-related gene scores for each patient and stratified them into the high and low MMP1 groups based on the median score. Patients in the high MMP1 group exhibited significantly shorter overall survival (OS) in both the training cohort (hazard ratio [HR] = 7.64, 95% CI: 6.19–9.42, *p* < 0.001) and the validation cohort (HR = 2.86, 95% CI: 1.54–5.29, *p* < 0.001) (Figure 6B). Consistent results were observed with the GBM model, where high MMP1 patients showed poor OS in both cohorts (Figure 6C). The 1-year survival prediction curves for the StepCox[forward] + GBM and GBM models demonstrated receiver operating characteristic (ROC) curves with areas > 0.7 in both the training and validation sets (Figure 6D). Integrating the StepCox[forward] + GBM and GBM models via univariate Cox regression and meta-analysis revealed that MMP1 + FIB-related genes are significant prognostic risk factors for tumor progression. The highly significant HR values (random-effects model HR = 5.49; fixed-effects model HR = 8.08) underscore their role as potential drivers of tumor progression (Figure 6E,F). These results demonstrate that the StepCox[forward] + GBM and GBM models constructed from MMP1 + FIB signature genes provide an effective predictive tool with robust specificity and sensitivity.

### 3.7. Spatial Transcriptomics and mIHC Reveal Distribution Heterogeneity of Immune Cells in the TME

**Figure 7:**
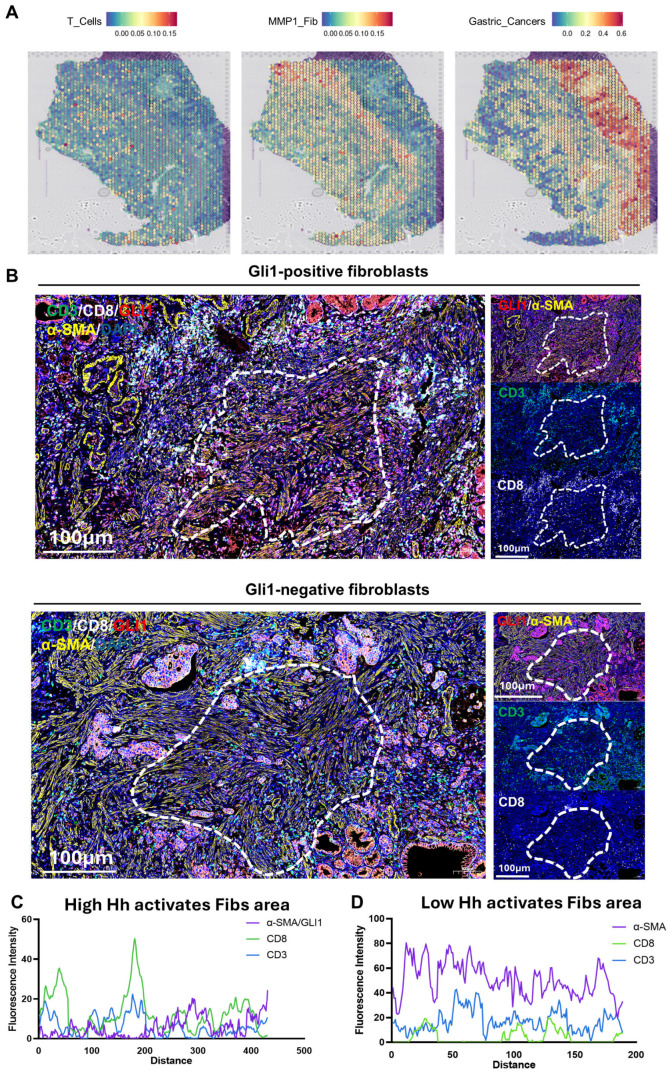
Observation of the spatial distribution of T cells, Hh-activated fibroblasts, and gastric cancer cells via spatial transcriptomics and mIHC. (**A**) Spatial transcriptomics data of T cells, MMP1 + FIB, and gastric cancer cells in gastric cancer pathological sections. (**B**) mIHC staining of 3 gastric cancer tissue sections. Cell nuclei: DAPI (blue fluorescence). T cells: CD3 (green fluorescence), CD8 (white fluorescence). Fibroblasts: α-SMA (yellow fluorescence). Molecular marker for Hedgehog signaling pathway activation: GLI1 (red fluorescence). (**C**) Fluorescence intensity distribution curve showing the spatial distribution relationship between GLI1-positive fibroblasts and T cells. (**D**) Fluorescence intensity distribution curve showing the spatial distribution relationship between GLI1-negative fibroblasts and T cells. FIB, fibroblast.

Furthermore, by integrating spatial transcriptomics data (GSE203612) with the results of mIHC experiments on clinical gastric cancer samples, this study further confirmed that the infiltration of MMP1 + FIB can remodel the TME in clinical patients. Analysis revealed significant spatial heterogeneity in the TME: T cells were primarily distributed in the left region, tumor cells occupied the right region, and MMP1 + FIB formed a physical barrier between the two (Figure 7A). Notably, T cell infiltration was significantly reduced in MMP1 + FIB-enriched regions, which is consistent with our previous findings—fibroblasts activated by the Hh signaling pathway can promote the deposition of collagen/fibronectin to form a dense ECM network. While enhancing the mechanical barrier function, this network further establishes an immunosuppressive microenvironment characterized by increased infiltration of M2-type macrophages and decreased numbers of anti-tumor immune cells (e.g., plasma cells, memory B cells). To validate this finding, this study performed mIHC experiments using formalin-fixed paraffin-embedded sections of gastric cancer specimens. All three samples were obtained from Zhejiang Provincial Hospital of Traditional Chinese Medicine (Supplementary Table S1). The results showed less T cell infiltration in the vicinity of GLI1-positive fibroblasts (GLI1 is the core gene in the Hedgehog signaling pathway), whereas more abundant T cell infiltration was observed around GLI1-negative fibroblasts (Figure 7B–D, Supplementary Figure S8A–C). It should be added that this study has previously confirmed that the infiltration level of MMP1 + FIB shows a positive correlation with the number of M2-type macrophages, and this cell subset can regulate myeloid cell function by secreting complement C3. Integrating the above results, this study proposes the following mechanistic model: MMP1 + FIB may induce the differentiation of M2-type macrophages via complement C3, thereby mediating T cell exhaustion and ultimately synergistically promoting the proliferation and metastasis of gastric cancer.

### 3.8. COMPLEMENT C3 Induces M2 Macrophage Differentiation to Promote Gastric Cancer Proliferation and Metastasis

**Figure 8:**
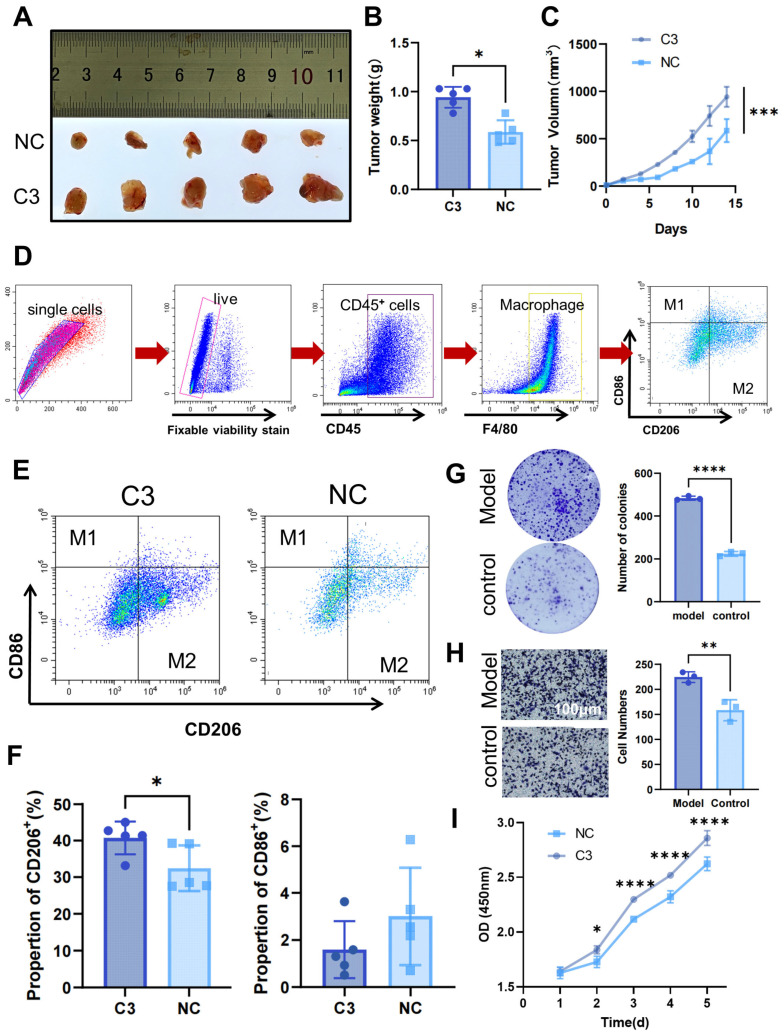
In vivo and in vitro experiments reveal that high Hh.score fibroblasts affect gastric cancer progression by secreting C3. Nude mice subcutaneously injected with C3 recombinant protein and MKN45 gastric cancer cells were used to assess (**A**) tumor growth and (**B**) tumor weight. (**C**) Tumor volume curves in xenograft models (*n* = 5 per group). (**D**) Flow sorting strategy. (**E**,**F**) Flow cytometry analysis of M1 and M2 macrophage proportions in tumor tissues. (**G**–**I**) Effects of macrophage-conditioned medium stimulated with C3 on MFC gastric cancer cells: (**G**) colony formation assay for clonogenic capacity, (**H**) Transwell chamber assay for invasion ability, and (**I**) CCK-8 assay for proliferation capacity. C3, C3 recombinant protein intervention group; NC, control group. * *p* ≤ 0.05; ** *p* ≤ 0.01; *** *p* ≤ 0.001; **** *p* ≤ 0.0001.

Building on these findings, we further investigated the mechanisms by which MMP1 + FIB remodel the tumor immunosuppressive microenvironment to promote gastric cancer proliferation and metastasis through in vitro and in vivo experiments. Previous studies have confirmed that MMP1 + FIB specifically secrete complement C3 by activating COMPLEMENT, thereby modulating myeloid cell function. Based on this evidence, the current study focused on complement C3-mediated regulation of myeloid cells and its underlying mechanisms. Given established evidence that Hh-activated fibroblasts secrete complement C3 [28,29], we specifically investigated whether complement C3 drives malignant progression via immunosuppressive microenvironment remodeling in gastric cancer. Nude mice were subjected to a one-week adaptive feeding period, after which the C3 group received subcutaneous injections of C3 recombinant protein (20 μg/kg) every other day for one week, while control mice were injected subcutaneously with an equal volume of physiological saline. Subcutaneous xenograft tumor models were then established using MKN45 gastric cancer cells. Tumor growth was assessed by measuring the tumor volume with calipers every two days, calculated using the following formula: volume = 1/2 × (major diameter) × (minor diameter)^2^. Mice were euthanized two weeks later. Experimental results showed that the tumor size and weight in the C3 group were significantly larger than those in the control group (Figure 8A–C). Flow cytometry was used to sort macrophages and analyze the proportions of M1 and M2 macrophages (M1/M2) in tumor tissues (Figure 8D). The results revealed a significant increase in the M2 macrophage proportions in the C3 group, while the M1 proportions exhibited a downward trend in the C3 group (Figure 8E,F). Further, co-culture of MFC gastric cancer cells with supernatant from C3 recombinant protein-stimulated RAW264.7 cells (conditioned medium) significantly enhanced gastric cancer cell proliferation and invasion (Figure 8G–I). Collectively, these findings confirm that high Hh.score_Fib promotes malignant phenotypes in gastric cancer by secreting COMPLEMENT C3, which induces myeloid cell differentiation into M2 macrophages, thereby facilitating tumor proliferation and metastasis.

## 4. Discussion

Hh is an evolutionarily highly conserved pathway that mediates signal transduction from the cell membrane to the nucleus [7]. Extensive research has demonstrated that aberrant activation of Hh drives the initiation and progression of various tumors, including GC [8]. Utilizing scRNA-seq, this study revealed significant activation of Hh signaling specifically within CAFs in the gastric cancer TME. Among these CAFs, the MMP1 + FIB subpopulation exhibited the highest level of Hh activation. Furthermore, we identified MMP1 + FIB as being specifically enriched within the gastric cancer TME. This finding carries significant implications, as compelling evidence indicates that CAFs promote malignant progression in GC by remodeling the TME, particularly through the establishment of an immunosuppressive niche. For instance, CAFs mediate immunosuppression via multiple mechanisms: secretion of immunosuppressive cytokines (e.g., TGF-β, IL-6, IL-10); recruitment and polarization of immunosuppressive immune cells (e.g., regulatory T cells, myeloid-derived suppressor cells, M2-type tumor-associated macrophages); and extracellular matrix remodeling that hinders cytotoxic T cell infiltration and function [30,31]. Given that MMP1 + FIB represent a distinct CAF subpopulation enriched in the gastric cancer TME and characterized by high Hh signaling activity, we hypothesize that this subpopulation may play a pivotal role in remodeling the local immune microenvironment, particularly by promoting immunosuppression. This reprogramming of the immune microenvironment likely represents a key mechanism through which MMP1 + FIB contributes to the aggressive phenotype of gastric cancer and ultimately leads to poor patient prognosis.

Further in-depth analysis in this study revealed that the MMP1 + FIB subpopulation localizes to the terminal stage of the fibroblast differentiation trajectory. Notably, as MMP1 + FIB cells undergo differentiation and maturation, the expression levels of core genes within Hh progressively increase, ultimately reaching their peak. This suggests a close, and potentially causal, link between the differentiation/maturation process of MMP1 + FIB and the aberrant activation of Hh signaling. Furthermore, MMP1 + FIB is functionally distinct from other fibroblast subpopulations, with its prominent characteristics including highly dysregulated metabolic activation, particularly a marked enhancement in amino acid and lipid metabolism. Concurrently, both the complement system and the TNF-α/NF-κB signaling pathway were significantly activated within this subpopulation. Studies indicate that aberrant activation of the complement system promotes GC progression through multiple mechanisms; for instance, the release of C3a and C5a can induce M2 macrophage polarization and suppress anti-tumor immunity; C5a attracts myeloid-derived suppressor cells to infiltrate GC tissue via C5aR1, inhibiting CD8+ T cell function and promoting immune evasion [32,33,34]. The TNF-α/NF-κB pathway shapes the immunosuppressive microenvironment in GC by recruiting immunosuppressive cells, suppressing effector T cell function, and upregulating checkpoint molecules and tolerogenic cytokines [35,36]. Additional research demonstrates that metabolic dysregulation in fibroblasts can contribute to poor tumor prognosis; for example, the secretion of unsaturated fatty acids (such as oleic acid) by fibroblasts can induce macrophage polarization toward the M2 phenotype, which secretes pro-tumorigenic factors like IL-10 and TGF-β [37]. We propose that MMP1 + FIB, characterized by aberrant Hh activation, likely promotes malignant phenotypes in GC—such as proliferation and metastasis—by remodeling the tumor immunosuppressive microenvironment.

Survival analysis and machine learning models consistently confirmed that high infiltration of MMP1 + FIB within the TME serves as an independent risk factor for poor prognosis in GC patients. Further molecular correlation analysis revealed that the abundance of MMP1 + FIB is significantly positively correlated with markers of tumor proliferation and metastasis, as well as immunosuppressive signatures. This substantiates our hypothesis that MMP1 + FIB contributes to the deterioration of patient prognosis by remodeling the immunosuppressive TME and directly or indirectly promoting GC proliferation and metastasis.

CellChat analysis further elucidated that MMP1 + FIB exhibits a significantly distinct signaling output pattern compared to PDGFA + FIB and IGLC2 + FIB. MMP1 + FIB specifically highly expresses and secretes complement component C3, establishing specific communication with myeloid cells via the C3–(ITGAX + ITGB2) ligand–receptor axis. Existing research clearly demonstrates that cleavage fragments generated upon C3 activation (C3a, C3b/iC3b) can bind to various receptors on myeloid cell surfaces. Notably, the integrin αXβ2 (CR4), composed of ITGAX (CD11c) and ITGB2 (CD18), is a key receptor of C3b/iC3b [28]. This axis not only mediates the recruitment and retention of myeloid cells into the TME but has also been reported to promote angiogenesis and epithelial–mesenchymal transition, thereby accelerating GC invasion and metastasis.

Based on these findings, we speculate that MMP1 + FIB, by specifically secreting C3 and activating the C3–(ITGAX + ITGB2) signaling axis, significantly influences the functional state of myeloid cells, collectively driving the remodeling of the tumor immunosuppressive microenvironment and ultimately promoting malignant progression and poor prognosis in GC. These findings were validated in bulk RNA-seq data. Notably, immune infiltration analysis demonstrated a significant positive correlation between the extent of MMP1 + FIB infiltration in tumor tissues and the abundance of M2-type tumor-associated macrophages. Conversely, a marked negative correlation was observed with the infiltration levels of anti-tumor immune cells, including T cells, B cells, and plasma cells. This phenotype is highly consistent with the known characteristics of an immunosuppressive microenvironment. Extensive research has elucidated that M2 macrophages directly suppress the activation, proliferation, and effector functions of T and B cells by releasing key factors such as TGF-β and IL-10 [38]. Furthermore, studies demonstrate that M2 macrophages secrete matrix metalloproteinases and fibronectin, promoting aberrant ECM remodeling and fibrosis, thereby forming a physical barrier that impedes the infiltration and function of effector immune cells like T and B cells into the tumor parenchyma [39].

Based on these results, we propose the following mechanism for MMP1 + FIB-driven GC progression: MMP1 + FIB specifically secretes complement C3; C3 directly recruits and influences myeloid cells via the C3–(ITGAX + ITGB2) axis; and more importantly, it likely induces the polarization of recruited myeloid cells (e.g., monocytes) into M2 macrophages with potent immunosuppressive capabilities. These M2 macrophages subsequently act synergistically through the aforementioned multiple mechanisms (secreting inhibitory factors, expressing checkpoint molecules, constructing physical barriers) to strongly suppress the infiltration and function of core effector cells of adaptive immunity, such as T and B cells. This ultimately leads to immune surveillance failure, promoting GC proliferation, invasion, metastasis, and poor patient prognosis.

Spatial transcriptomic analysis combined with the mIHC results from clinical GC samples further confirmed that the spatial localization and distribution characteristics of MMP1 + FIB significantly negatively regulate the infiltration of anti-tumor immune defense cells into the tumor parenchyma. In vitro and in vivo functional experiments provided direct evidence of the above mechanism: exogenous complement C3 treatment effectively induced the differentiation of myeloid cells toward the M2 macrophage phenotype. Moreover, the resulting M2 macrophages exhibited significant pro-tumorigenic activity in in vivo models, including directly promoting the proliferation, invasion, and metastasis of gastric cancer cells.

Collectively, our study demonstrates that fibroblasts with aberrant Hh activation can remodel the immunosuppressive tumor microenvironment in GC, which is significantly associated with poor patient prognosis. While this research provides valuable insights, we acknowledge several inherent limitations. First, we identified significant Hh activation occurring during MMP1 + FIB differentiation but did not validate whether Hh activation induces the differentiation of fibroblasts into MMP1 + FIB. Additionally, although we established a robust clinical predictive model, the future development of specific inhibitors targeting MMP1 + FIB is crucial. Strategies such as nanoparticle or liposome-based delivery systems enabling precise targeting of both the specific MMP1 protein structure and key Hh pathway components warrant exploration.

This study identified aberrant activation of Hh in CAF within the TME. Specifically, the MMP1 + FIB subpopulation exhibited significantly higher levels of Hh activation compared to other CAF subgroups and was preferentially enriched in the gastric cancer TME. Mechanistically, this subpopulation promotes the differentiation of myeloid cells into M2 macrophages through the secretion of complement C3, thereby driving the proliferation and metastasis of gastric cancer.

## 5. Conclusions

This study uncovers a novel mechanism by which fibroblasts with highly activated Hh accelerate gastric cancer progression: these fibroblasts specifically secrete complement C3, thereby driving the polarization of M2-type macrophages and the formation of an immunosuppressive microenvironment, and ultimately, accelerating the progression of gastric cancer. The enrichment of MMP1 + FIB is significantly associated with poor prognosis in patients with gastric cancer; moreover, the regulation of myeloid cells mediated by MMP1 + FIB via the C3–(ITGAX + ITGB2) axis plays a central role in the immune evasion of gastric cancer. This finding not only deepens our understanding of the heterogeneity of the gastric cancer microenvironment and the mechanisms underlying the formation of immunosuppression but also provides potential targets for therapeutic strategies targeting the Hh-MMP1 + FIB-C3 axis.

## Data Availability

The datasets presented in this study can be found in online repositories. The names of the repository/repositories and accession number(s) can be found below: GSE163558, GSE183904, and GSE203612(GEO); TCGA-STAD, TCGA-FPKM(TCGA).

## Supplementary Materials

The following supporting information can be downloaded at https://www.mdpi.com/article/10.3390/cancers17193164/s1, Figure S1. Single-cell RNA sequencing analysis of dataset GSE163558. Figure S2. Single-cell RNA sequencing analysis of dataset GSE183904. Figure S3. Association of MMP1 + FIB with malignant progression in gastric cancer. Figure S4. Differentiation dynamics of Hh-related genes. Figure S5. CellChat analysis reveals cell–cell communication roles of PDGFA + FIB and IGLC2 + FIB in the gastric cancer TME. Figure S6. GSEA identifies significantly enriched gene sets in patients with high MMP1 scores. Figure S7. Correlation between MMP1 + FIB infiltration levels and immune cell infiltration in patients. Figure S8. Spatial distribution of Hh pathway-activated fibroblasts and T cells in gastric cancer tissues. Table S1: Clinical information of patient.

## Abbreviations

The following abbreviations are used in this manuscript:

CAFs	Cancer-associated fibroblasts
DEGs	Differentially expressed genes
GC	Gastric cancer
Hh	Hedgehog signaling pathway
Hh.score	Hh-related gene scores
High_MMP1	High MMP1 signature gene score groups
IGLC2 + FIB	IGLC2-positive fibroblasts
Low_MMP1	Low MMP1 signature gene score groups
mIHC	Multiplex immunofluorescence
MDSCs	Myeloid-derived suppressor cells
MMP1 + FIB	MMP1-positive fibroblasts
PDGFA + FIB	PDGFA-positive fibroblasts
TME	Tumor microenvironment
TIME	Tumor immune microenvironment
