# Supplementary material for: M2 Macrophage Polarization Mediated by Complement C3 from Hedgehog-Activated Fibroblasts Establishes an Immunosuppressive Niche in Gastric Cancer
Source: Cancers (Basel). 2025 Sep 29;17(19):3164. doi: 10.3390/cancers17193164 (PMC12523485; doi:10.3390/cancers17193164)
Supplement: Supplementary file 1 [file cancers-17-03164-s001.zip › cancers-3884586-supplementary.pdf]

Supplementary Material

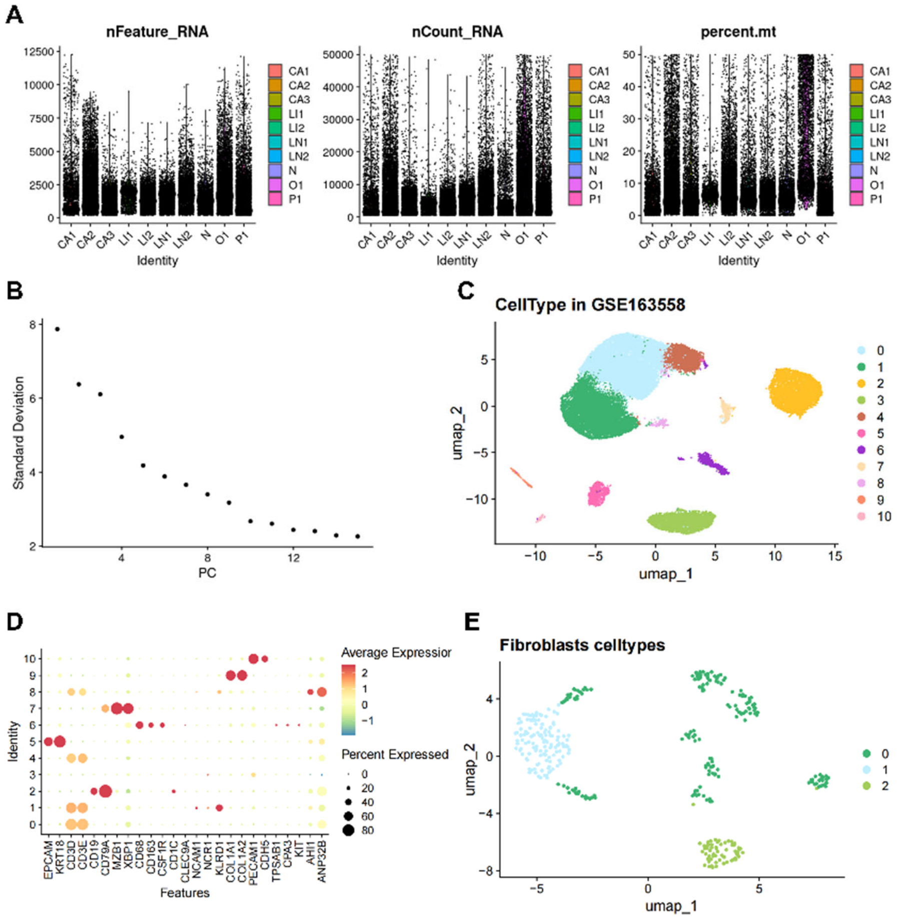

Figure S1. Single-cell RNA sequencing analysis of dataset GSE163558. (A) Violin plots show the distribution of the number of RNA features, RNA counts, and the percentage of mitochondrial RNA in different sample groups. (B) Walking plot of the relationship between principal components and standard deviation in single-cell analysis. (C) UMAP clustering shows the distribution of the identified cell clusters. (D) Bubble plot shows the marker genes of 10 main cell types. (E) UMAP clustering shows the distribution of the identified fibroblast cell clusters.

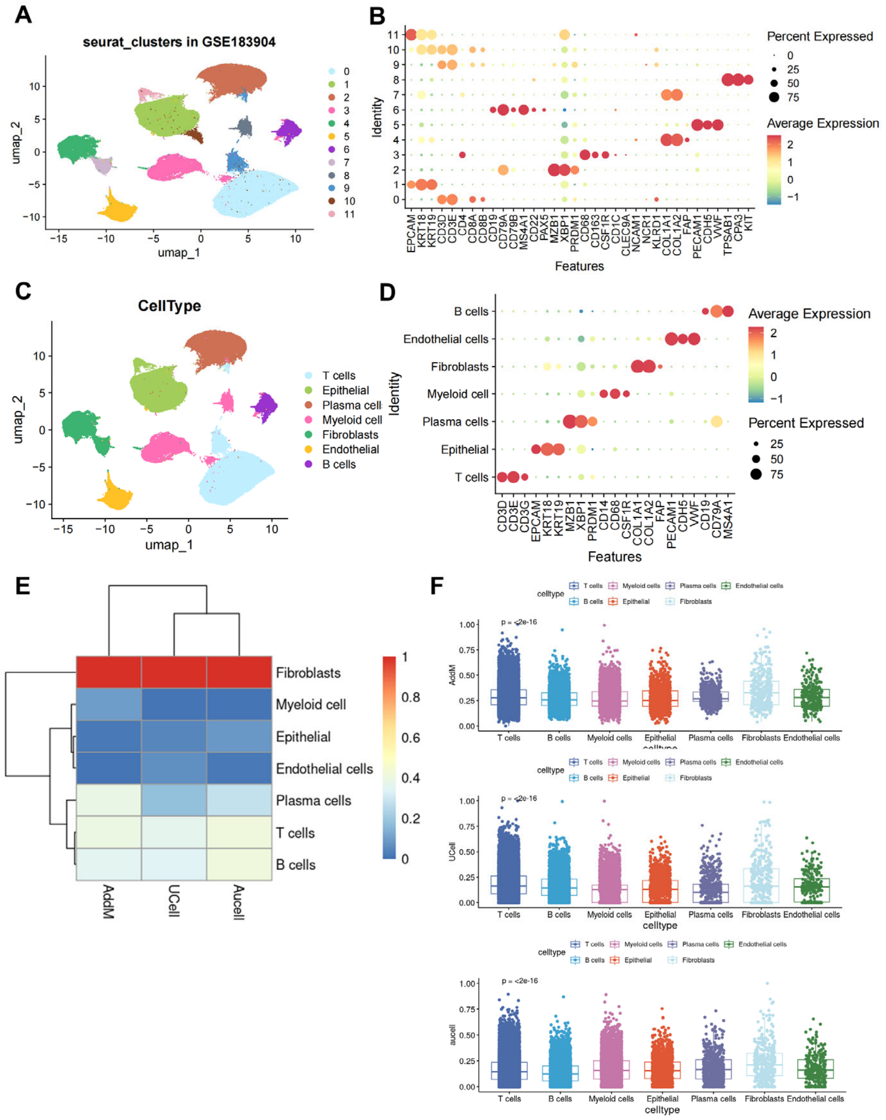

Figure S2. Single-cell RNA sequencing analysis of dataset GSE183904. (A) UMAP clustering shows the distribution of the identified cell clusters. (B) Bubble plot shows the marker genes of 11 main cell types. (C) UMAP clustering shows their respective cell type annotations. (D) Bubble plot shows the marker genes of seven main cell types.(E)UMAP showing the heatmap of activation levels of the Hh gene set in seven cell subsets in the GSE183904 dataset. (F) Box plots show the differences in Hedgehog (Hh) pathway enrichment scores among seven cell types (based on three independent analytical algorithms: AddModuleScore, UCell, and AUCell).

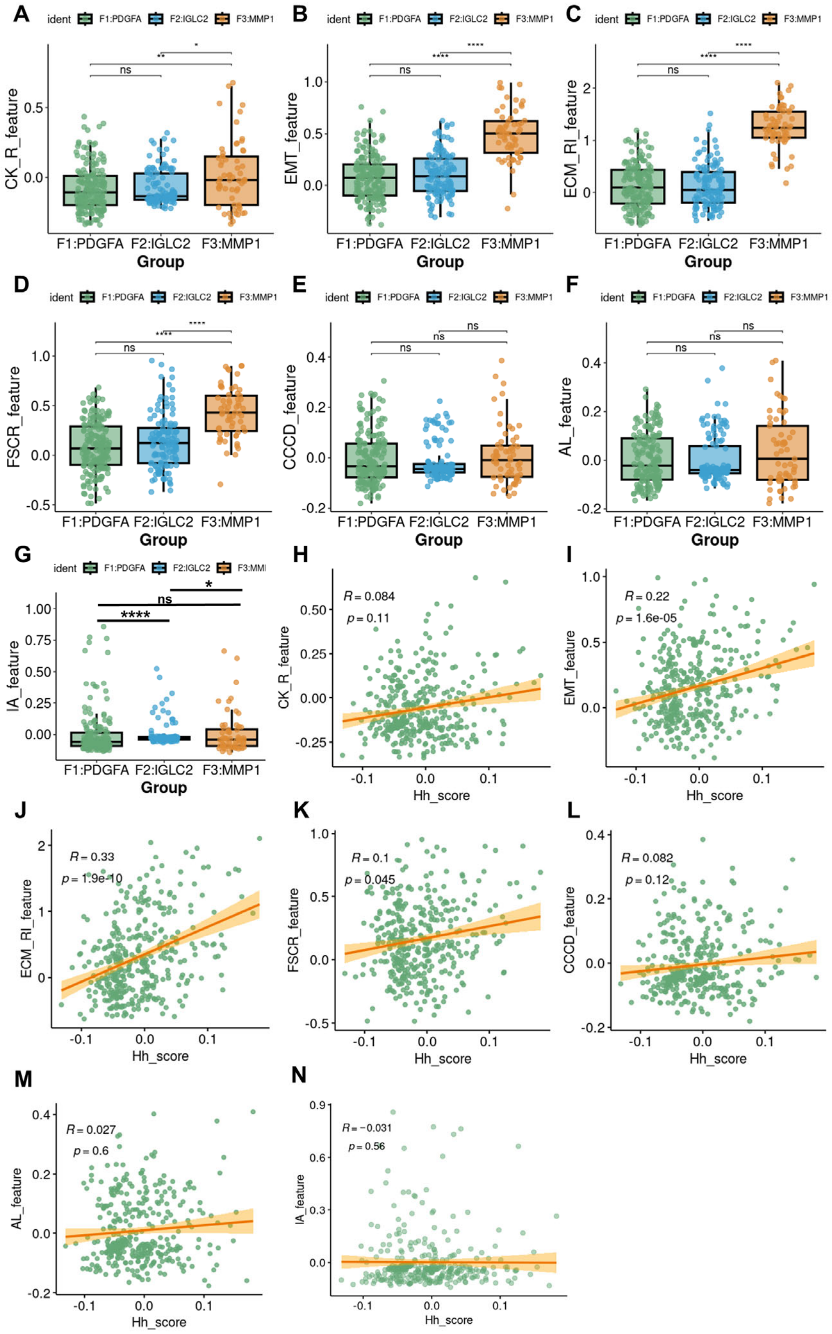

Figure S3. Association of MMP1+FIB with malignant progression in gastric cancer. (A-G) Inter-group differential analyses show MMP1+FIB and PDGFA+FIB, IGLC2+FIB in (A) inflammatory chemotaxis, (B) epithelial-mesenchymal transition, (C) ECM remodeling and invasion, (D) fibroblast and stromal cell regulation, (E) cell cycle imbalance, (F) angiogenesis and lymphangiogenesis, (G) Immune activation (H-N) Correlation analysis shows Hh activation degree of fibroblasts with (H) inflammatory chemotaxis, (I) epithelial-mesenchymal transition, (J) ECM remodeling and invasion, (K) fibroblast and stromal cell regulation, (L) cell cycle imbalance, (M) angiogenesis and lymphangiogenesis, (N) Immune activation.

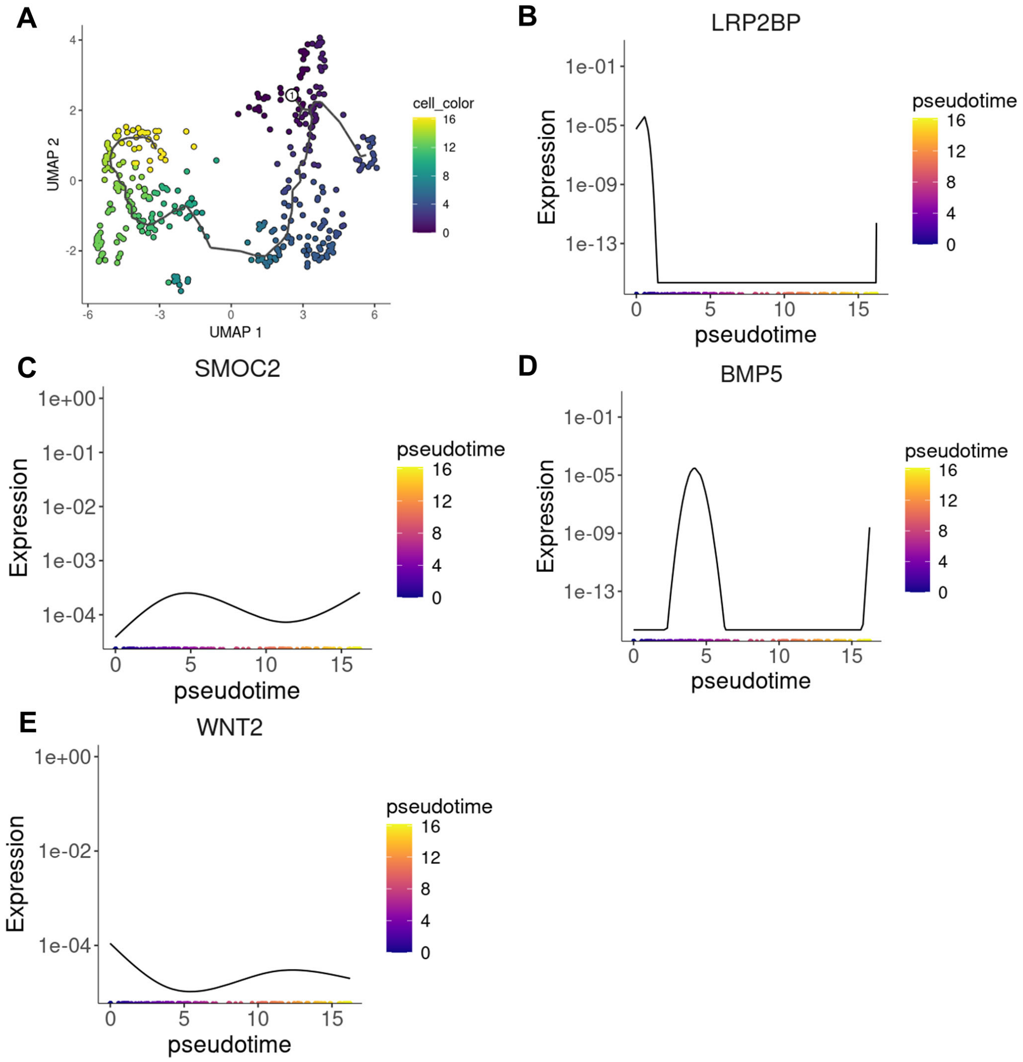

Figure S4. Differentiation dynamics of Hh-related genes. (A) UMAP plot shows the pseudotime trajectories of different fibroblast subpopulations. (B-E) Differentiation trajectory plots of Hh-related genes in pseudotime analysis.

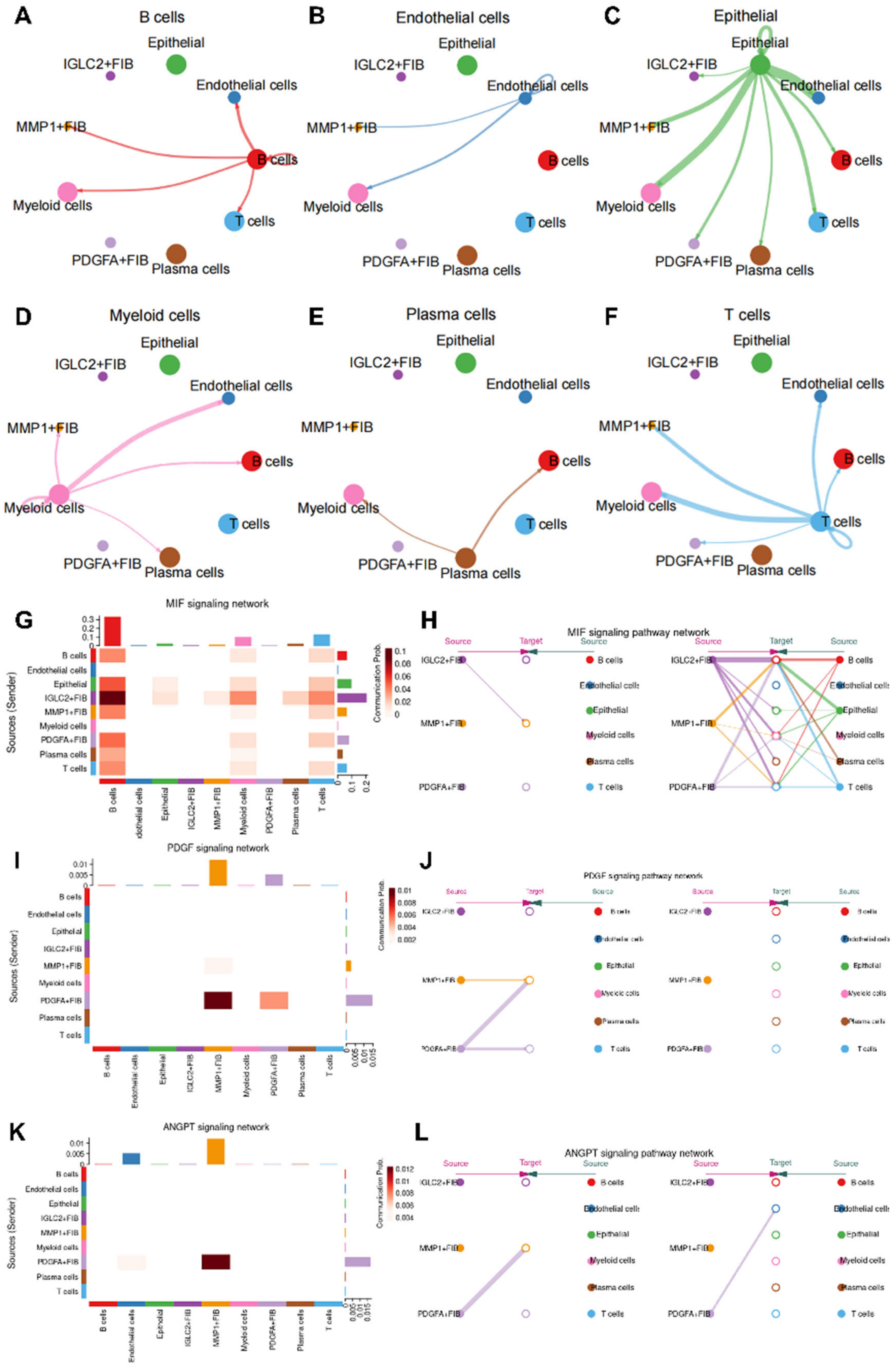
A
B cells
B
Endothelial cells
C
Epithelial
D
Myeloid cells
E
Plasma cells
F
T cells
Epithelial
IGLC2+FIB
Endothelial cells
MMP1+FIB
B cells
Myeloid cells
T cells
PDGFA+FIB
Plasma cells
G
MIF signaling network
Sources (Sender)
Communication Prob.
H
MIF signaling pathway network
Source
Target
I
PDGF signaling network
J
PDGF signaling pathway network
K
ANGPT signaling network
L
ANGPT signaling pathway network

Figure S5. CellChat analysis reveals cell-cell communication roles of PDGFA+FIB and IGLC2+FIB in the gastric cancer TME. (A–F) Cell-cell communication networks among all cell types in the gastric cancer TME. (G) Heatmap depicting the relative strength of the MIF signaling pathway network per cluster, including incoming and outgoing signaling patterns; color gradients represent scaled interaction strength. (H) Hierarchical plot showing the signaling cascade of the MIF pathway. (I) Heatmap illustrating the relative strength of the PDGF signaling pathway network per cluster, including incoming and outgoing signaling patterns; color gradients indicate scaled interaction strength. (J) Hierarchical plot displaying the signaling cascade of the PDGF pathway network. (K) Heatmap presenting the relative strength of the ANGPT signaling pathway network per cluster, including incoming and outgoing signaling patterns; color gradients reflect scaled interaction strength. (L) Hierarchical plot demonstrating the signaling cascade of the ANGPT pathway.

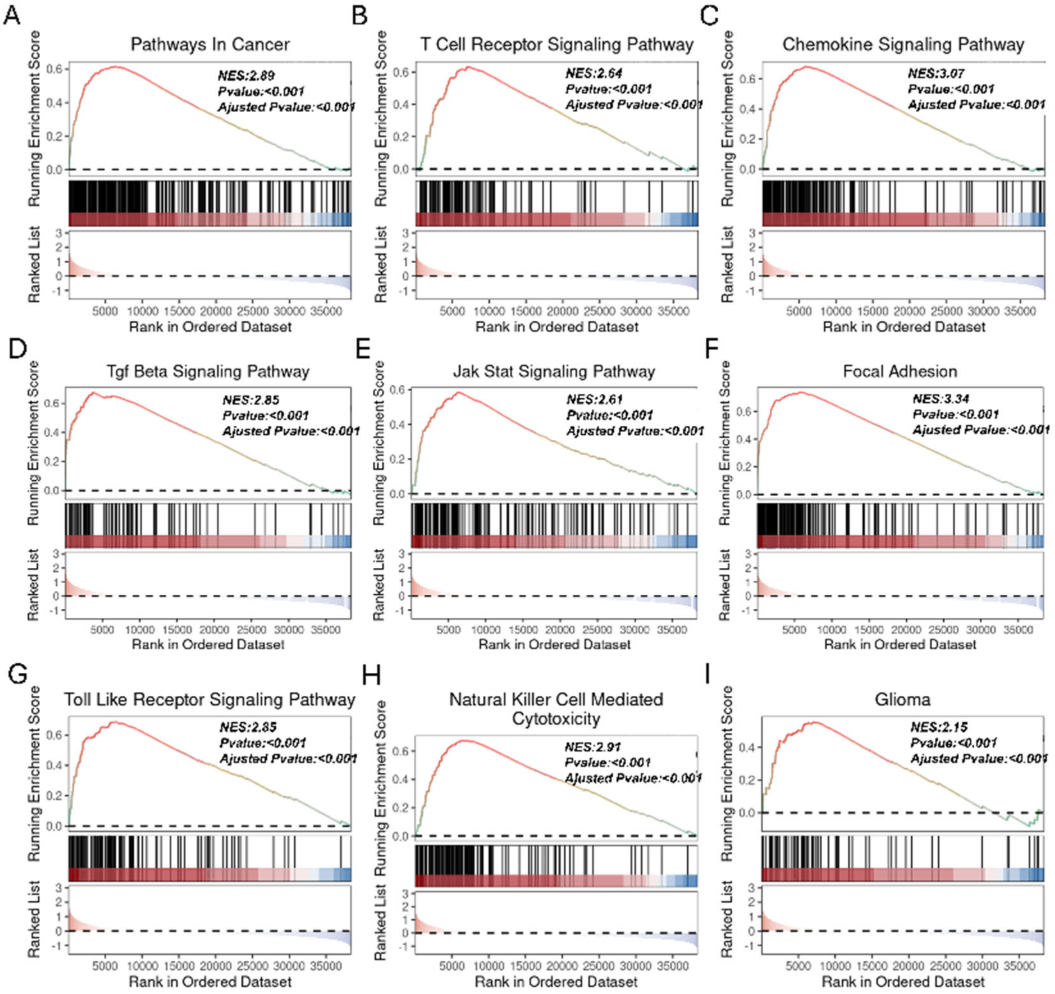

Figure S6. GSEA identifies significantly enriched gene sets in patients with high MMP1 scores. (A–I) GSEA reveals significant enrichment of immunosuppression-related gene sets in high MMP1-score patients.

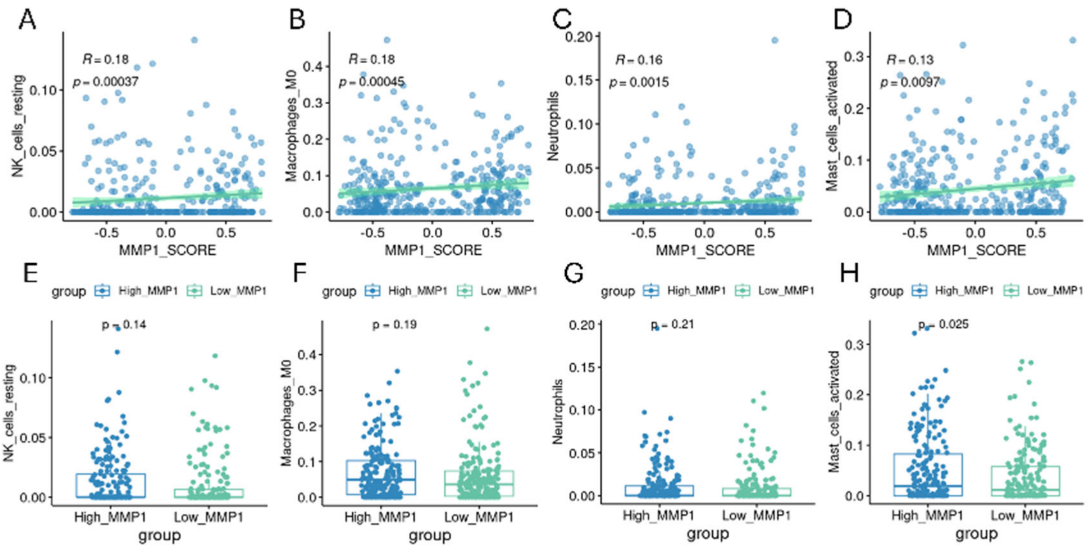

Figure S7. Correlation between MMP1+FIB infiltration levels and immune cell infiltration in patients. (A–D) Scatter plots showing correlations between MMP1+FIB infiltration levels and infiltration scores of resting NK cells, M0 macrophages, neutrophils, and activated mast cells, respectively. (E–H) Box plots demonstrating differences in infiltration scores of resting NK cells, M0 macrophages, neutrophils, and activated mast cells between High_MMP1 and Low_MMP1 patient groups.

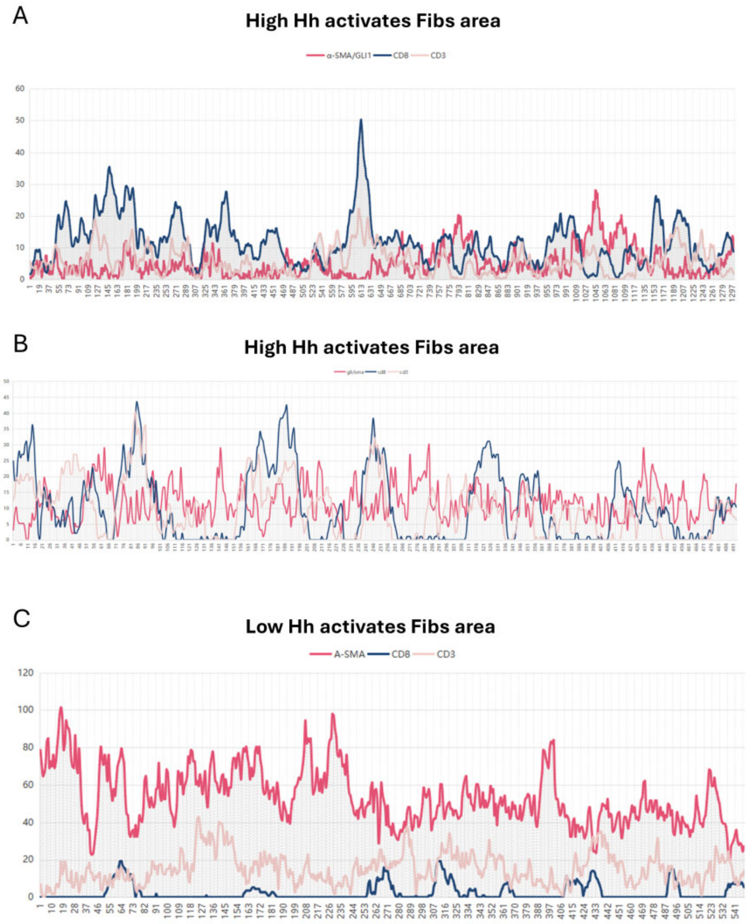

Figure S8. Spatial Distribution of Hh Pathway-Activated Fibroblasts and T Cells in Gastric Cancer Tissues (A-C) Fluorescence intensity distribution curves showing the spatial distribution relationship between GLI1-positive fibroblasts and T cells
